# Root-Associated Fungal Communities From Two Phenologically Contrasting Silver Fir (*Abies alba* Mill.) Groups of Trees

**DOI:** 10.3389/fpls.2019.00214

**Published:** 2019-03-05

**Authors:** Tina Unuk, Tijana Martinović, Domen Finžgar, Nataša Šibanc, Tine Grebenc, Hojka Kraigher

**Affiliations:** ^1^Department of Forest Physiology and Genetics, Slovenian Forestry Institute, Ljubljana, Slovenia; ^2^Institute of Microbiology of the Czech Academy of Sciences, Prague, Czechia; ^3^Biotechnical Faculty, Department of Agronomy, University of Ljubljana, Ljubljana, Slovenia

**Keywords:** host phenology, stand age, root-associated fungi, silver fir, fungal community

## Abstract

Root-associated fungal communities are important components in ecosystem processes, impacting plant growth and vigor by influencing the quality, direction, and flow of nutrients and water between plants and fungi. Linkages of plant phenological characteristics with belowground root-associated fungal communities have rarely been investigated, and thus our aim was to search for an interplay between contrasting phenology of host ectomycorrhizal trees from the same location and root-associated fungal communities (ectomycorrhizal, endophytic, saprotrophic and pathogenic root-associated fungi) in young and in adult silver fir trees. The study was performed in a managed silver fir forest site. Twenty-four soil samples collected under two phenologically contrasting silver fir groups were analyzed for differences in root-associated fungal communities using Illumina sequencing of a total root-associated fungal community. Significant differences in beta diversity and in mean alpha diversity were confirmed for overall community of ectomycorrhizal root-associated fungi, whereas for ecologically different non-ectomycorrhizal root-associated fungal communities the differences were significant only for beta diversity and not for mean alpha diversity. At genus level root-associated fungal communities differed significantly between early and late flushing young and adult silver fir trees. We discuss the interactions through which the phenology of host plants either drives or is driven by the root-associated fungal communities in conditions of a sustainably co-naturally managed silver fir forest.

## Introduction

In forest ecosystems, tree roots are colonized by various ecto- and/or endo-mycorrhizal fungi as well as by other root-associated fungi. Ecto- and endomycorrhizal fungi in particular are recognized as potential drivers of nutrient mobilization processes and hormonal regulation of the mycorrhizal plants (Gogala, 1991; Kraigher et al., 1991, 2008; Toju et al., 2013). There are two main functional groups of fungi in forest soils: (1) free-living saprotrophic fungi and (2) fungi that are symbiotically associated with plant roots (Smith and Read, 2008). The root-associated fungal symbiosis is of key importance as fungi influence plant productivity and plant community diversity, protect roots from grazing and pathogens, as well as connect plants belowground via a hyphal network allowing the movement of resources among coexisting plants (van der Heijden et al., 2015).

In general, ectomycorrhizal (ECM) fungi enhance the growth and survival of their host plant through improving nutrient uptake in exchange for photosynthetically derived carbon (Kraigher, 1996). Although mycorrhizal fungi associate with a majority of plants, each individual plant benefits most from a unique root-associated fungal community (Smith and Gianinazzi-Pearson, 1988). The functional compatibility depends on the species and strain of the fungus, and the species and population of the host (Gianinazzi-Pearson, 1984). Consequently, changes in the mycorrhizal community composition may influence plant nutrient status, growth and development (Wardle et al., 2004). Different factors can influence ECM community structure, including host plant species composition, stand age, habitat conditions, and edaphic factors (Smith et al., 2002). Several authors have observed a strong effect of tree age on fungal communities, as litter quality and understory vegetation in young stands differ markedly from older stands (Last et al., 1987). Younger stands also differ in nutrient and water dynamics, soil microclimate and litter quantity and quality (Kyaschenko et al., 2017). Due to rapid growth in early developmental stages, young trees generally have the potential to sequester a larger amount of carbon (Croft et al., 2014), mainly because of carbon allocation to ECM root tips (Wallander et al., 2010).

Besides ectomycorrhizal fungi, root tips are also frequently associated with endophytic fungi, which indicates possible competitive colonization of roots between endophytic and ectomycorrhizal fungi (Jumpponen and Trappe, 1998). However, while ECM fungi generally associate with a narrow range of hosts (Sato et al., 2007), root-endophytic fungi are not as host-specific and have a broader host range (Walker et al., 2011). The first report about another type of root colonization by septate endophytes was reported by Gallaud (1905) on *Allium sphaerocephalum* L. and *Ruscus aculeatus* L. Subsequently named dark septate entophytes (DSE), the DSE have only recently received more attention, and there is still much uncertainty about the roles of DSE. Although the interfaces formed by DSE differ from the conventional types of interfaces observed in mycorrhizal symbiosis, some studies (Jumpponen et al., 1998; Newsham, 1999; Barrow and Aaltonen, 2001) have shown that under some environmental conditions, DSE can enhance host growth and nutrient uptake; however, it remains unanswered as to whether DSE can increase hosts’ fitness in the long term (Jumpponen, 2001). Although it has been suggested that some DSE genera are mycorrhizal (Jumpponen, 2001), due to unusual fungal structures and lack of demonstration of host nutritional benefit from the symbiosis DSE associations have not been considered to be mycorrhizal (Jumpponen, 2001; Lukešova et al., 2015).

Host-symbiont relationships may be among others directly influenced by the phenology of hosts. It has been suggested that the host’s phenology affects the activity of ECM fungi through the seasonal photosynthate allocation to the roots, and throughout seasonal changes of carbohydrate availability (Egli et al., 2010; Sato et al., 2012; Hupperts et al., 2017). Conversely, mycorrhizal fungi may affect host phenology through providing the carbon at a time when demand is high and photoassimilates are not yet available (Courty et al., 2010). The composition and turnover of ECM fungi may affect the set of functions performed by an ECM community, which can generate feedback on changes in host phenology (Hupperts et al., 2017).

As processes in host and ECM fungi phenology need further clarification, we are going to anticipate the relations in our study solely as an interplay between the host tree phenology and age and the root-associated fungal community. In this study, we describe the community composition structure of root-associated fungi in a silver-fir dominated forest, in terms of (1) tree host phenology, (2) tree age and (3) a combination of phenology and tree age. We hypothesize that ECM and other root-associated fungal species richness (alpha diversity) differ based on phenological characteristics and tree age, and is expected to be higher in association with adult and earlier flushing silver fir trees. For adult stands it has been reported to be associated with more diverse and species richer fungal communities compared to young stands, while for earlier flushing silver fir tree we expect to find higher fungal species richness due to earlier availability of photosynthates (Hypothesis 1). As plant and microbial processes vary throughout the season, which has a strong impact on carbon and nitrogen availability, we suspect that ECM and other non-ECM root-associated fungal community structure will have significantly different species turnover among trees with contrasting phenology and based on tree age (Hypothesis 2), and that consequently we will observe a significant difference in ECM and other non-ECM root-associated fungal genera abundances among the four analyzed groups.

## Materials and Methods

### Description of Study Site and Sampling

The study was performed in 16.47 hectares of a forest site at Lehen na Pohorju (Pohorje, Slovenia, 46.33N 15.20E, 469-611 m a.s.l.). dominated by native silver fir (*Abies alba* Mill.), with the age range of trees surrounding the plots between 35 and 120 years. The last major harvest at the study site took place between 1985 and 1992. Phenology stages of each individual tree or seedling in natural regenerating centers were assessed based on a standardized protocol (LIFEGENMON protocols; in prep), where the time of budbreak and flowering date were monitored consecutively in 2017. Individual trees with extreme phenologies (earliest/latest budbreak and flowering, the difference being from 3 to 5 weeks in a single year) were selected and further separated into a group of adult (dominant trees, at least 80 years old, with a minimum diameter at breast height of 31 cm) and young trees (in understory/shade, maximum 50 years old and with a maximum diameter at breast height of 5 cm). Six silver fir trees and six soil samples for each phenology × age combination were analyzed, resulting in a total of 24 soil samples in total collected according to the sampling protocol described in Kraigher (1996). All soil samples with silver fir fine roots were sampled in August 2017. To avoid sampling of roots from other adult trees, the soil samples were collected approximately 1 m from the trunk, whereas individual silver fir trees were at least 5–7 m apart to minimize sampling in the same common mycorrhizal network (*sensu* Baar et al., 1999; Taylor and Bruns, 1999; Varela-Cervero et al., 2015; Chen et al., 2016). Plots of young silver fir seedlings were selected at a distance of at least 8–10 m. Soil samples were taken to 20 cm deep (including organic layers) using soil corers. Individual samples were placed in plastic bags and stored at 6°C before processing. From each sample all roots were removed from soil and gently washed to remove all the remaining soil particles attached to the roots. For further processing, roots with diameter less than 2 mm (e. g., fine roots *sensu* Železnik et al., 2007, 2016) were selected and freeze dried at −92°C for 48 h. After freeze drying, roots were homogenized into a fine powder using a Retsch mixer mill (Retsch, Haan, Germany).

### Molecular Analyses

DNA was extracted from 50 mg of individual soil sample root powder using Reagent DNeasy Plant Mini Kit (Qiagen, Hilden, Germany) following manufacturer’s instructions. Additional DNA purification was done with Geneclean Turbo Kit (MP Biomedicals, Solon, OH, United States). Twenty-four differentially barcoded gITS7 forward primer and ITS4 reverse primer (Ihrmark et al., 2012) were used to amplify the ITS2 region of fungal ribosomal DNA. PCR mix contained 2.5 μl 10× polymerase buffer, 1.5 μl bovine serum albumin, 0.5 μl of PCR Nucleotide Mix (10 mM), 1 μl of each primer, 0.75 μl of polymerase (2 U μl^−1^; Pfu DNA polymerase: Dynazyme DNA polymerase, 1:24) and 1 μl of template DNA. PCRs were carried out in three replicates per sample, using the following thermocycling conditions: initial 5 min at 94°C, followed by 30 cycles at 94°C for 30 s, 56°C for 30 s, 72°C for 30 s and final cycle of 7 min at 72°C. To confirm the success of amplification, each PCR product was run on 1% agarose gel. Triplicates from the same root sample were then merged and purified with MinElute PCR Purification Kit (Qiagen, Hilden, Germany). Finally, the DNA concentration was determined by Qubit 2.0. Fluorometer (Life Technologies, Carlsbad, CA, United States) and equimolar concentrations were sequenced on Ilumina MiSeq (300 bp paired end).

### Bioinformatics

The sequencing data generated from MiSeq run was processed using the pipeline SEED v2.0 (Větrovský and Baldrian, 2013). Pair-end reads were merged using FASTQ-join (Aronesty, 2011). The ITS2 region was further extracted with ITSx 1.0.11 (Bengtsson-Palme et al., 2013). Chimeric sequencing among the amplicons pool was removed using USEARCH (Edgar, 2013). Sequences were clustered at 97% similarity threshold using VSEARCH (Rognes et al., 2016), aligned using MAFFT 7.222 (Katoh et al., 2009) and blasted against UNITE (Kõljalg et al., 2013) and NCBI^1^ databases for ITS-based classification. Sequences identified as non-fungal were discarded, as well as sequences that remained unclassified at a kingdom or family level. Finally, the following criteria for a database match were applied: sequence similarity >92%, which represent approximate cut-off value at genus level (Porras-Alfaro et al., 2014) and a BLAST e-value <1e-40. ECM OTUs were separated from non-ECM fungi based on the genus status using the references Rinaldi et al. (2008) and Tedersoo et al. (2010), while non-ECM fungi were further grouped based on their ecology described in literature (Sigler et al., 2000; Ganley et al., 2004; Geml et al., 2005; Hambleton and Sigler, 2005; Crous et al., 2007; Voriškova and Baldrian, 2013; Lodge et al., 2014; Hilszczanska, 2016; Bödeker et al., 2016; Vaz et al., 2017) into endophytes, saprotrophs and pathogens (Supplementary Table S1). None of the fungi remained unassigned.

### Diversity and Statistical Analysis

To check for the possible effect of variable sequences sampling depth on analyzed groups of silver fir, differences in number of fungal sequences per individual group were tested using the Kruskal-Wallis test from statistical package “stats” (R Core Team, 2016), which revealed no significant differences in fungal sequences number among all four analyzed groups (*P* = 0.5641). Spatial patterns of diversity were examined by alpha diversity indices (assessed by Hill’s numbers; Hill, 1973) to determine whether the fungal community was correlated with age and phenology of trees. We used the most commonly used diversity indices: OTU richness, inverse Simpson’s index, which calculates the proportional abundance of each species and is interpreted as equivalent to OTU evenness, and inverse Berger-Perker index, which calculates the proportional abundance of the most abundant species and can be interpreted as OTU dominance.

Observed differences in fungal community composition between sites (beta diversity) were quantified with a pairwise permutational multivariate analysis of variance (pairwise PERMANOVA) based on the Bray-Curtis distance matrix. Differences in overall fungal community composition were visualized using non-metric multidimensional scaling analysis (NMDS) ordinations using the Bray-Curtis distance matrix.

One-way ANOVA with Tukey contrasts was used to examine the difference in alpha diversity among the four groups (adult late and early flushing trees and young late and early flushing trees). The homogeneity of variance assumptions was checked with a Levene test (*P* = 0.102).

Multivariate generalized linear models (MV-GLMs; Wang et al., 2012) were used to examine the differences in ECM and endophytic, saprophytic and pathogenic genera abundances between phenologically different adult and young silver fir trees. Multivariate and unadjusted univariate *P*-values were obtained by Wald tests, both using 10,000 Monte Carlo permutations. For visualization of MV-GLM results, endophytic, saprotrophic and pathogenic fungal genera with abundance higher than 1% were included in the plot.

All statistical, diversity and community analyses were conducted on a normalized number of OTUs using R statistical language version 3.4.4 with standard R libraries (R Core Team, 2016). The community analyses were performed with PERMANOVA on the Bray-Curtis distance matrix, and visualized with NMDS on the Bray-Curtis distance, using the specific package ‘vegan’ (Oksanen et al., 2018). One-way ANOVA with Tukey contrasts for alpha diversity analysis was performed using statistical package ‘stats’ (R Core Team, 2016) and MV-GLMs with the package ‘mvabund’ (Wang et al., 2012). Differences in ECM, endophytic, saprotrophic and pathogenic abundances between phenologically different adult and young silver fir trees was visualized using the statistical package “ggplot2” (Wickham, 2016).

## Results

### Fungal Community Structure Varies With Phenology in the Young but Not in the Old Trees

The overall analysis was based on a total of 367,294 fungal ITS2 sequences that remained after the removal of non-fungal sequences and unclassified sequences at a kingdom or family level and length trimming following the Illumina MiSeq sequencing. The overall dataset was divided into ECM OTUs and endophytic, saprotrophic and pathogenic root-associated fungal OTUs.

**FIGURE 1:**
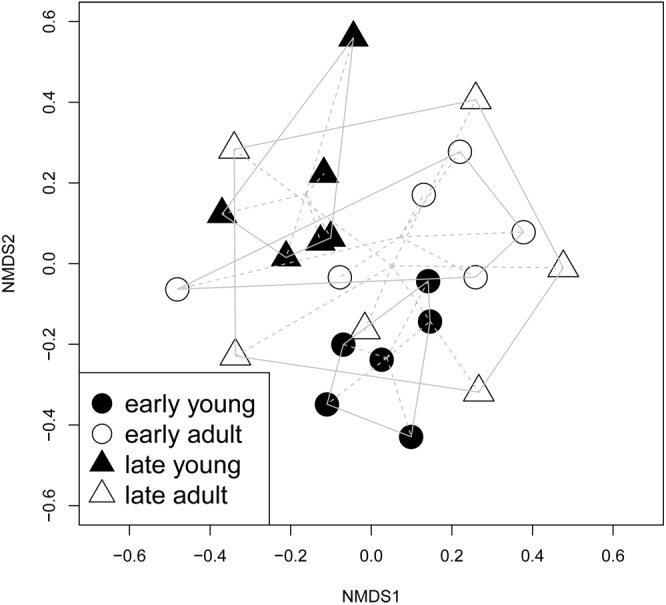
Ectomycorrhizal (ECM) fungal community ordination plot based on communities from early/late flushing and adult/young silver fir trees, calculated on a Bray-Curtis distance with a non-metric multidimensional scaling analysis (NMDS) ordination. Presented are centroids of each groups of samples. The ECM community significantly differs between early and late flushing young silver fir trees (PERMANOVA, *P* = 0.003), but not between early and late flushing adult silver fir trees (PERMANOVA. *P* = 0.9910).

The NMDS showed significantly different ECM communities between early and late flushing young trees (PERMANOVA, *P* = 0.003), while ECM communities of adult silver trees partially overlapped, with no significant differences between early and late flushing trees (PERMANOVA, *P* = 0.991) (Figure 1).

**FIGURE 2:**
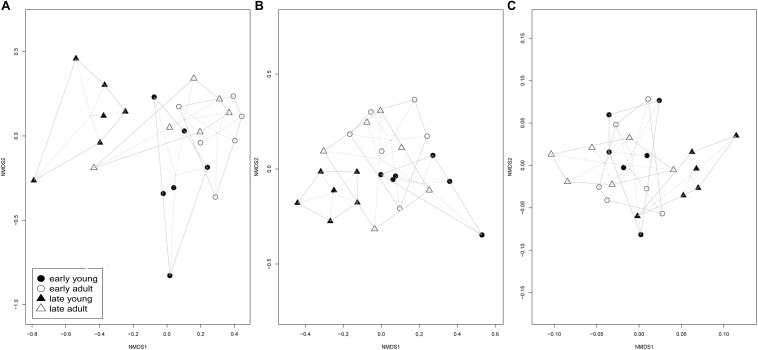
Endophytic **(A)**, saprotrophic **(B)** and pathogenic **(C)** fungal community ordination plots based on communities from early/late flushing and adult/young silver fir trees, calculated on a Bray-Curtis distance with a non-metric multidimensional scaling analysis (NMDS) ordination. Presented are centroids of each groups of samples. **(A)** Endophytic fungal community significantly differs between early and late flushing young silver fir trees (PERMANOVA, *P* = 0.006), but not between early and late flushing adult silver fir trees (PERMANOVA, *P* = 0.532). **(B)** The saprotrophic fungal community also significantly differed between early and late flushing young silver fir trees (PERMANOVA, *P* = 0.004), but not between early and late flushing adult silver fir trees (PERMANOVA, P = 0.401). **(C)** The same was revealed for the pathogenic fungal community, which significantly differed between early and late flushing young silver firs (PERMANOVA, *P* = 0.004), but not between early and late flushing adult silver fir trees (PERMANOVA, *P* = 0.935).

Also, in the case of ecologically different non-ECM root fungal communities (Figure 2), NMDS showed significantly different endophytic (PERMANOVA, *P* = 0.006), saprotrophic (PERMANOVA, *P* = 0.004) and pathogenic root fungal communities (PERMANOVA, *P* = 0.004) between early and late flushing young silver fir trees, while all three groups of non-ECM fungal communities of adult silver fir trees overlapped, with no significant differences between early and late flushing trees (PERMANOVA endophytes, *P* = 0.532; PERMANOVA saprotrophs, *P* = 0.401; PERMANOVA pathogens, *P* = 0.935).

**FIGURE 3:**
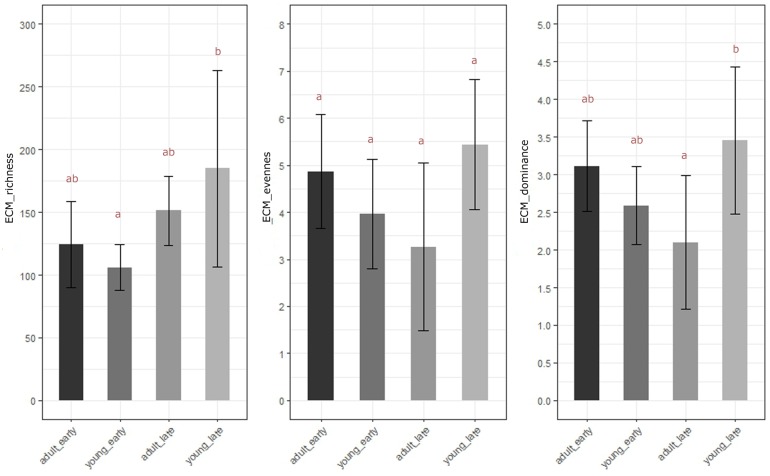
Mean alpha diversity with +/- standard deviation of root-associated fungi as displayed as richness, evenness and dominance (Hill, 1973) of ectomycorrhizal (ECM) fungi, based on community analyses from early/late flushing and young/adult trees. There is a significant difference in OTU richness between early and late flushing young silver fir trees (ANOVA, *P* = 0.033) and in dominance of the ECM fungal community between late flushing young silver fir trees and late flushing adult trees (ANOVA, *P* = 0.030). Different letters mark significantly different results (Tukey HSD test, *P* < 0.05).

Alpha diversity results coincide with differences in ECM observed in NMDS plots and supported with PERMANOVA. One-way ANOVA with Tukey contrasts conducted on mean alpha diversity among four groups of samples revealed significantly higher ECM fungal richness (Figure 3) in late flushing young silver fir trees compared to earlier flushing young trees (ANOVA, *P* = 0.0336), whereas for ECM fungal richness of adult silver fir trees only the influence of interaction between phenology and age was observed. We did not observe significant differences in mean evenness in ECM communities (ANOVA, *P* > 0.063), whereas the ECM community sampled from late flushing young silver fir trees showed significantly less dominance compared to late flushing adult silver fir trees (ANOVA, *P* = 0.030).

There was no significant difference (*P* > 0.106) in mean alpha diversity of the endophytic, saprotrophic or pathogenic root-associated fungal community.

### Genus-Level Diversity

Among the 3,753 ECM fungal OTUs found in 24 root samples, 2772 (73.8%) were identified to genus and 2150 (57.2%) to species level. Among the 1681 non-ECM root-associated fungal OTUs found, 1316 (78.2%) were identified to genus and 706 (41.9%) to species. In order of abundance, the most abundant ECM fungi were assigned to genera *Russula*, *Lactarius*, *Elaphomyces*, *Tylospora* and *Cenococcum*, and represented 50.5% of all sequences. For the non-ECM community, in order of abundance, endophytic fungi assigned to genera *Meliniomyces*, *Oidiodendron Phialocephala* and saprophytic fungi *Luellia* were among the most abundant root-associated fungi and represented 27.5% of all sequences.

**FIGURE 4:**
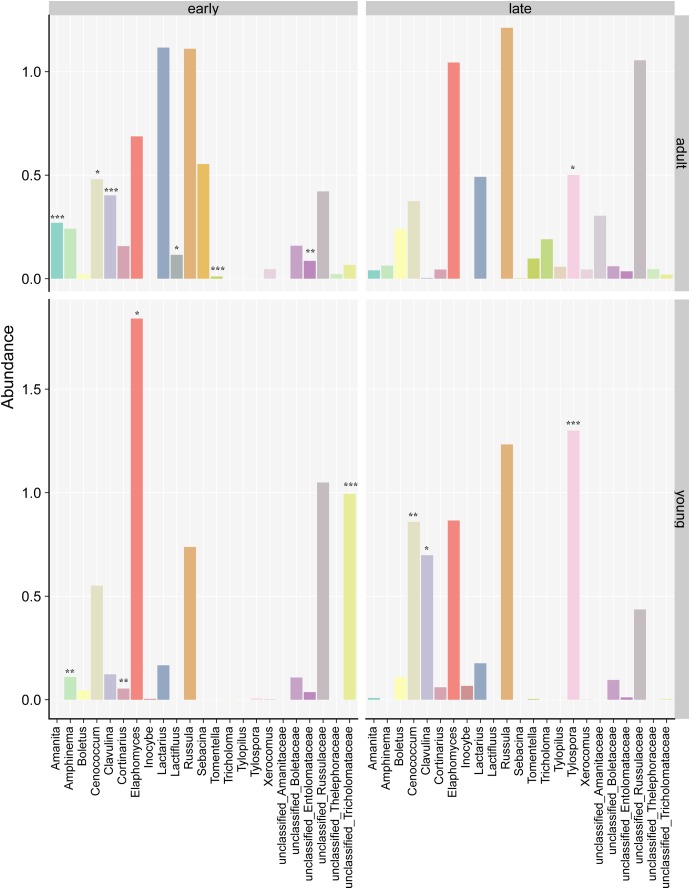
Abundances of individual ectomycorrhizal (ECM) fungal genera on silver fir in relation to phenology and age. Different signs mark significantly higher genera abundances between early and late flushing trees separately for adult and young silver fir trees (Multivariate generalized linear models, ^∗∗∗^*P* < 0.001; ^∗∗^*P* < 0.01; ^∗^*P* < 0.05). For all genera abundances results, see Supplementary Table S1.

For adult silver fir trees, MV-GLM showed significant more abundant (Supplementary Table S1) ECM fungal genera *Amanita*, *Cenococcum*, *Clavulina*, *Lactifluus*, *Sebacina* and unclassified genera assigned to the order Entolomataceae (MV-GLM, *P* < 0.037) with early flushing adult silver fir trees compared to late flushing adult trees (Figure 4). In the case of young silver fir trees, we observed significantly more abundant ECM genera *Amphinema*, *Cortinarius*, *Elaphomyces* and unclassified genera assigned to Tricholomataceae (MV-GLM, *P* < 0.037) with early flushing silver fir trees, in contrast to genera *Cenococcum*, *Clavulina*, and *Tylospora* (MV-GLM, *P* < 0.011) which were significantly more abundant in association with late flushing silver fir trees.

**FIGURE 5:**
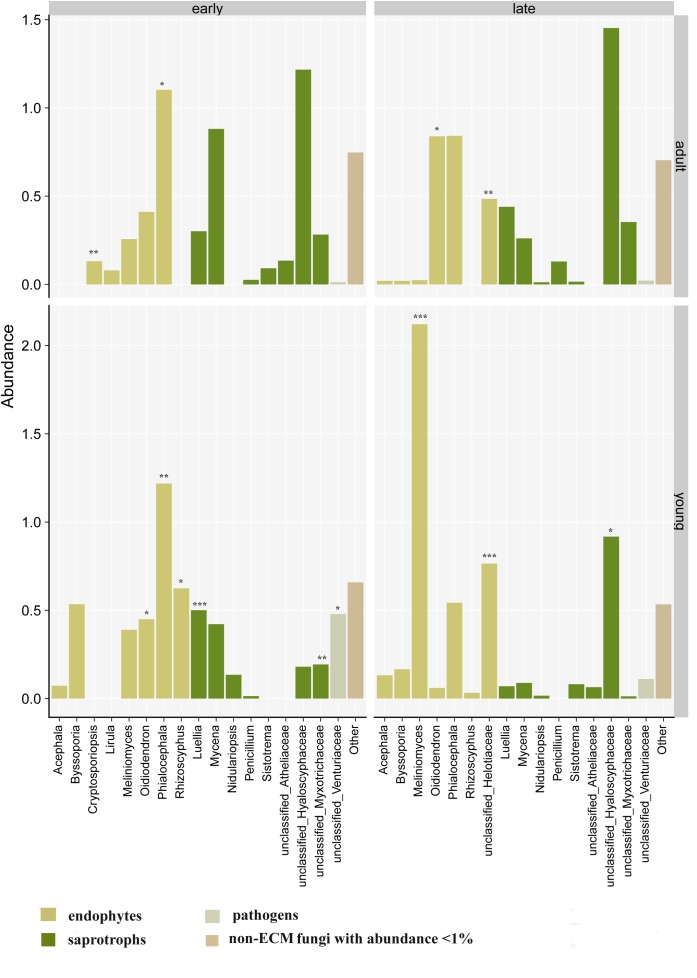
Abundances of individual endophytic, saprophytic and pathogenic root-associated fungal genera of silver fir in relation to phenology and age; operational taxonomic units with abundance lower than 1% were included in analysis and statistics but excluded from the plots. Different signs mark significantly higher genera abundances between early and late flushing trees separately for adult and young silver fir trees (Multivariate generalized linear models, ^∗∗∗^*P* < 0.001; ^∗∗^*P* < 0.01; ^∗^*P* < 0.05). For all genera abundances results, see Supplementary Table S1.

In the case of non-ECM root-associated fungal genera (Figure 5) associated with adult silver fir trees, MV-GLM showed significant differences in abundances of endophytic fungal genera, namely *Cryptosporiopsis*, *Oidiodendron*, *Phialocephala* and of unclassified genera from the order Helotiaceae (MV-GLM, *P* < 0.049) (Supplementary Table S1). For young silver fir trees, MV-GLM revealed significant differences in endophytic root-associated fungal genera *Oidiodendron*, *Phialocephala*, and *Rhizoscyphus*, and the saprophytic root-associated fungal genus *Luellia* and in unclassified genera assigned to Myxontrichaceae, whose abundance was higher in early flushing young trees. Compared to those, the endophytic genus *Meliniomyces* and unclassified genera from endophytic Helotiaceae and saprotrophic Hyaloscyphaceae (MV-GLM, *P* < 0.043) were significantly more abundant in association with late flushing silver fir trees. Root-associated fungi assigned to unclassified Venturiaceae were the only pathogenic fungi with abundance higher than 1% and with significant differences between early and late flushing young silver fir trees (MV-GLM, *P* = 0.025).

## Discussion

Correlation of aboveground plant phenological characteristics with belowground root-associated fungal communities in natural forest ecosystems has rarely been tested in scientific studies mainly due to expected complex interactions in the rhizosphere and complicated assessment of potential effects between above- and belowground parts of symbionts (Igiehon and Babalola, 2018). We have confirmed the potential interplay of tree phenology and the ectomycorrhiza (ECM), endophytic, saprophytic and pathogenic fungal community belowground as the community on young trees differed between earliest and latest flushing trees from the same location. This only partially confirms the hypothesis on the significant interplay between aboveground plant phenological characteristics with belowground root-associated fungal communities. The non-significant interplay between the phenology of adult silver fir trees and the ECM fungal community could be a consequence of more equal carbon capture of mature trees, and of carbohydrates exchange through already well-established common mycelial networks, which reduce mycorrhizal community composition turnover (Fernandez-Toiran et al., 2006; Simard, 2009; Croft et al., 2014; Kyaschenko et al., 2017).

The age of the stand was the second factor expected to significantly affect the ectomycorrhizal and non-ECM root-associated fungal genera on silver fir under uniform site conditions. We expected higher abundance of ECM and endophytic root fungi OTUs in older silver fir trees, as many authors have noted lower-diversity ECM and with ECM fungi associated endophytic fungal community on younger seedlings, which increases as the root systems and stands develop (Peter et al., 2001; Korkama et al., 2006; Fernandez-Toiran et al., 2006; Twieg et al., 2007). The outcome of the community comparison between young and old silver fir trees from natural stands was the opposite of that expected. Many fungi were present throughout forest stand development although their abundance and dominance changed with time or disturbance. We expected to find higher richness of ECM OTUs in early flushing silver fir trees, as due to the earlier availability of photosynthates and carbon allocation to the roots ectomycorrhizas may become active earlier. Counter to this hypothesis, we found significant higher diversity of ECM OTUs in late flushing silver fir trees. A significant difference was also revealed in ECM dominance for late flushing silver fir trees, whereas late flushing adult silver fir trees had higher dominance of OTUs than late flushing young silver fir trees, suggesting that “faster” or “earlier” ECM species prevail in flushing adult trees, preventing other species from colonizing roots, and that the production of signaling molecules, such as phytohormones, influences the onset of an earlier bud-bursting. We also expected to find higher abundances of endophytic and saprotrophic fungi in association with younger silver fir trees, as was the case for non-ECM root-associated fungal communities reported to show a higher species richness in younger seedlings, suggesting that especially endophytic root-associated fungi may be pioneering colonizers of young tree seedlings in the studied conditions (Mandyam and Jumpponen, 2005), whereas we found no significant difference in mean alpha diversity of the endophytic, saprophytic and pathogenic root-associated fungal community.

The significant ECM community differences between early and late flushing young silver fir trees were also reflected in abundances of ECM at the genus level. In association with early flushing young silver fir trees, *Elaphomyces* dominated with a significant difference from late flushing trees. On the other hand, in late flushing young silver firs, *Cenoccocum*, *Clavulina*, *Russula* and *Tylospora* co-dominated with similar abundances. In general, occurrence of *Tylospora* in late flushing adult trees was unexpected, as this genus associates more frequently with young trees, as recoded by Korkama et al. (2006), Wallander et al. (2010), and Kyaschenko et al. (2017), who suggested that *Tylospora* might be a fast-growing species whose abundance declines when forests mature, and when other ECM fungi become dominant. We might explain the observed shifts in ECM community composition of young silver fir trees based on phenology by linking it to carbon cycling processes, as proposed by Kaiser et al. (2010), Clemmensen et al. (2014), and Hupperts et al. (2017). Shifts in ECM community structure between early and late flushing trees may also be a result of a direct link between ECM fungi and host photosynthetic activity (Pena et al., 2010). In phenologically different adult trees, although some significant differences in abundances of ECM genera have been observed, early and late flushing adult trees still show similar overall ECM community structure, with no significant community composition turnover, and with dominance of *Elaphomyces*, *Lactarius* and *Russula*.

Ectomycorrhiza on silver fir has been frequently studied in recent decades (Agerer, 2008). The abundant ECM fungal OTUs identified in this study are generally known as common members of fungal communities of temperate and boreal forest in Europe, including members of the genera *Cenoccocum*, *Russula*, *Lactarius*, etc. (Rudawska et al., 2016). Besides the already listed genera, members of the genera *Clavulina*, *Elaphomyces*, *Amanita*, *Boletus*, *Sebacina* and *Tylospora* were already found to be associated and typical in silver fir stands (Comandini et al., 1998; Wazny, 2014; Rudawska et al., 2016; Arguelles-Moyao et al., 2017; Ważny and Kowalski, 2017). Yet their exact role in framing the plant partner phenology remains unexplored.

Our study also revealed a pattern of community changes for non-ECM fungi with different ecology (endophytic, saprotrophic and pathogenic root-associated fungi) in a silver fir dominated forest, similar to that of the ECM community. Significant interplay between tree phenology and all non-ECM ecological groups of root-associated fungal community was confirmed only for young silver fir trees, but not also for adult silver fir trees, whereas despite some significant differences in individual genera abundances, overall no significant difference in non-ECM root-associated fungal community composition between these two groups was observed. In contrast to the ECM fungal community of early flushing young silver fir trees where *Elaphomyces* dominated, several non-ECM genera with similar abundances prevailed, for example endophytic fungi assigned to genera *Oidiodendron*, *Rhizoscyphus*, and *Phialocephala* and saprophytic root-associated fungi assigned to *Luellia* and unclassified genera assigned to Myxontrichaceae. In association with late flushing young trees *Meliniomyces* stood out with a significantly higher abundance compared to other non-ECM root-associated fungi. Most of the detected non-ECM root-associated fungal genera associated with silver fir roots were classified as endophytic fungi, for which co-occurrence with mycorrhizal fungi has already been reported (i.e., by Kernaghan and Patriquin, 2011; Porras-Alfaro and Bayman, 2011; Toju et al., 2013). *Abies* species are known to associate with endophytic fungi from Helotiales (Sieber, 2007), whereas Kernaghan and Patriquin (2011) also recorded *Phialocephala*, *Oidiodendron*, *Meliniomyces*, host-specific *Cryptosporiopsis*, genera from the Helotiaceae family and the saprophytic fungal genera *Penicillium* and *Mycena*. Those findings coincide with our results, whereas the mentioned endophytic genera were among the most abundant non-ECM fungi isolated from root samples of silver fir analyzed in this study. The revealed pattern of co-occurrence of ECM fungi and root endophytes suggests that ECM and root endophytes may have an array of possible ecological interactions in roots, which may be mutualistic or commensal rather than completely neutral (Toju et al., 2013).

The significant interplay between the host tree phenology and root-associated fungal community was confirmed for young silver fir trees, but not for adult trees. All four analyzed groups of root-associated fungal communities significantly differed between early and late flushing young silver fir trees, which only partly confirmed Hypothesis 1, as there was no significant difference in root-associated communities of phenologically contrasting adult silver fir trees. There was also no significant difference in root-associated fungal communities between adult and young silver fir trees in general, as we hypothesized. Mean alpha diversity analysis confirmed significant difference in fungal richness and dominance only for the ECM fungal community of silver fir trees, whereas there was no significant difference in mean alpha diversity for the other three groups of non-ECM root-associated fungal communities. At the genus level, significant differences of some ECM, endophytic, saprophytic and pathogenic fungal genera between phenologically contrasting adult and young silver fir trees were confirmed, which is in line with previously cited results. This study is one of the few in which root-associated fungal communities are linked to the host tree phenology, which gave us interesting results and an insight into the possible effect of root-associated fungi on host phenology and vice versa, since a significant interplay between root-associated fungal communities and phenologically contrasting host trees was confirmed.

## Data Availability

The analyzed dataset for this study can be found in the MG-RAST public database, under ID number mgm4821901.3 (Link: https://www.mg-rast.org/linkin.cgi?metagenome=mgm4821901.3).

## Author Contributions

This work is a part of the Ph.D. thesis of TU, under the mentorship of TG. TU, TG, and HK set up the experiments. TU done the laboratory analyses, performed the NGS with the assistance of TM, contributed to site selection and site parameters assessed by DF, and performed the statistical analysis with the contribution of NŠ. TU prepared the manuscript. TM, DF, NŠ, TG, and HK refined the manuscript.

## Conflict of Interest Statement

The authors declare that the research was conducted in the absence of any commercial or financial relationships that could be construed as a potential conflict of interest.

## Footnotes

1 https://www.ncbi.nlm.nih.gov/
