# Supplementary material for: Root-Associated Fungal Communities From Two Phenologically Contrasting Silver Fir (Abies alba Mill.) Groups of Trees
Source: Front Plant Sci. 2019 Mar 5;10:214. doi: 10.3389/fpls.2019.00214 (PMC6413537; doi:10.3389/fpls.2019.00214)
Supplement: Supplementary file 1 [file Table_1.docx]

Supplementary Material

# Supplementary Table

Table S1: Multivariate generalized linear models (MV-GLM) of ECM, endophytic, saprophytic and pathogenic root-associated genera abundances between phenologically contrasting silver fir trees based on tree age. Multivariate and unadjusted univariate P-values were obtained by Wald tests, both using 10,000 Monte Carlo permutations (P < 0.001***, P < 0.01**, P < 0.05*).

| **Genus** | **Mycorrhizal status** | **Genera abundances** | | **Reference** |
| --- | --- | --- | --- | --- |
|  |  | **Adult silver fir** | **Young silver fir** |  |
| *Acephala* | endophytic | 0.1509 | 0.1596 | Hilszczanska 2016 |
| *Amanita* | ECM | 0.0009 *** | 0.2548 | Rinaldi et al. (2008), Tedersoo et al. (2010) |
| *Amphinema* | ECM | 0.7222 | 0.0051 ** | Rinaldi et al. (2008), Tedersoo et al. (2010) |
| *Boletus* | ECM | 0.2374 | 0.3009 | Rinaldi et al. (2008), Tedersoo et al. (2010) |
| *Byssoporia* | endophytic | 0.1256 | 0.6953 | Kernaghan and Patriquin 2011, Hilszczanska 2016 |
| *Cenococcum* | ECM | 0.0355* | 0.0082 ** | Rinaldi et al. (2008), Tedersoo et al. (2010) |
| *Clavulina* | ECM | 0.0009 *** | 0.0165 * | Rinaldi et al. (2008), Tedersoo et al. (2010) |
| *Cortinarius* | ECM | 0.1 | 0.0056 ** | Rinaldi et al. (2008), Tedersoo et al. (2010) |
| *Cryptosporiopsis* | endophytic | 0.0081 ** | 1 | Kernaghan and Patriquin 2011, Hilszczanska 2016 |
| *Elaphomyces* | ECM | 0.4485 | 0.0106 * | Rinaldi et al. (2008), Tedersoo et al. (2010) |
| *Lactarius* | ECM | 0.2084 | 0.7998 | Rinaldi et al. (2008), Tedersoo et al. (2010) |
| *Lactifluus* | ECM | 0.0371 * | 1 | Rinaldi et al. (2008), Tedersoo et al. (2010) |
| *Lirula* | endophytic | 0.7002 | 0.6511 | Ganley et al. 2004 |
| *Luellia* | saprotrophic | 0.3964 | 0.0007 *** | Bödeker et al. 2016 |
| *Meliniomyces* | endophytic | 0.1690 | 0.0000 *** | Kernaghan and Patriquin 2011, Hilszczanska 2016 |
| *Mycena* | saprotrophic | 0.1561 | 0.5375 | Bödeker et al. 2016 |
| *Nidulariopsis* | saprotrophic | 1 | 0.5977 | Geml et al. 2005 |
| *Oidiodendron* | endophytic | 0.0491 * | 0.0491 * | Kernaghan and Patriquin 2011, Hilszczanska 2016 |
| *Penicilium* | saprotrophic | 0.1189 | 0.4753 | Vaz et al. 2017 |
| *Phialocephala* | endophytic | 0.0252 * | 0.0090 ** | Kernaghan and Patriquin 2011, Hilszczanska 2016 |
| *Rhizoscyphus* | endophytic | 1 | 0.015 * | Hambleton and Sigler 2005 |
| *Russula* | ECM | 0.1224 | 0.6681 | Rinaldi et al. (2008), Tedersoo et al. (2010) |
| *Sebacina* | ECM | 0.0003 *** | 1 | Rinaldi et al. (2008), Tedersoo et al. (2010) |
| *Sistotrema* | saprotrophic | 0.2971 | 0.1220 | Voriškova and Baldrian 2013 |
| *Tomentella* | ECM | 0.6737 | 0.1255 | Rinaldi et al. (2008), Tedersoo et al. (2010) |
| *Tylospora* | ECM | 0.0133 * | 0.0000 *** | Rinaldi et al. (2008), Tedersoo et al. (2010) |
| *Xerocomus* | ECM | 0.8438 | 0.2686 | Rinaldi et al. (2008), Tedersoo et al. (2010) |
| unclassified Atheliaceae | saprotrophic | 0.2176 | 0.1146 | Lodge et al. 2014 |
| unclassified Boletaceae | ECM | 0.3727 | 0.2845 | Rinaldi et al. (2008), Tedersoo et al. (2010) |
| unclassified Entolomataceae | ECM | 0.0053 ** | 0.2123 | Rinaldi et al. (2008), Tedersoo et al. (2010) |
| unclassified Helotiaceae | endophytic | 0.0014 ** | 0.0001 *** | Kernaghan and Patriquin 2011, Hilszczanska 2016 |
| unclassified Hyaloscyphaceae | saprotrophic | 0.8559 | 0.0431 * | Voriškova and Baldrian 2013 |
| unclassified Myxotrichaceae | saprotrophic | 0.4630 | 0.00189 ** | Sigler et al. 2000 |
| unclassified Russulaceae | ECM | 0.5669 | 0.9059 | Rinaldi et al. (2008), Tedersoo et al. (2010) |
| unclassified Thelephoraceae | ECM | 0.8268 | 0.59344 | Rinaldi et al. (2008), Tedersoo et al. (2010) |
| unclassified Tricholomataceae | ECM | 0.1120 | 0.0000 *** | Rinaldi et al. (2008), Tedersoo et al. (2010) |
| unclassified Venturiaceae | pathogenic | 0.3320 | 0.0254 * | Crous et al. 2007 |
